# Pharmacokinetic and Pharmacodynamic Effects of Polyclonal Antibodies against SARS-CoV2 in Mice

**DOI:** 10.3390/v15010123

**Published:** 2022-12-30

**Authors:** Aruni Jha, Melanie Doyle-Eisele, David Revelli, Trevor Carnelley, Douglas Barker, Shantha Kodihalli

**Affiliations:** 1 Emergent BioSolutions Canada Inc., Winnipeg, MB R3T 5Y3, Canada; 2 Lovelace Biomedical Research Institute, Albuquerque, NM 87108, USA

**Keywords:** COVID-19, pharmacokinetics, pharmacodynamics, mouse model

## Abstract

Despite ongoing vaccination efforts to prevent SARS-CoV-2 infections, treatment tools are still necessary to address the ongoing COVID-19 pandemic. We report here that COVID-HIGIV, a human immunoglobulin product for treatment of COVID-19, provided a significant survival benefit in SARS-CoV-2 infected transgenic mice compared to controls. COVID-HIGIV also has similar pharmacokinetic profiles in healthy and SARS-CoV-2 infected mice over time after intravenous administration, with identical or comparable Tmax, Cmax, AUC_0–∞_ and Cl. AUC_0–last_ increased and mean residence time, T_1/2_, and Vd reduced in infected animals compared to healthy animals. These data suggest that COVID-HIGIV may be an effective treatment for SARS-CoV-2 infection when given early after exposure.

## 1. Introduction

Severe acute respiratory syndrome coronavirus-2 (SARS-CoV-2) is the cause of an ongoing worldwide COVID-19 pandemic that has affected 223 countries and territories. Over 600 million confirmed cases and 6 million deaths have been reported worldwide [1]. The introduction of vaccines has had a positive impact on the pandemic; however, millions of new cases and thousands of associated fatalities have continued to occur, primarily due to non-accessibility of the vaccine in low-to-middle income countries and people’s choice of not taking the vaccine due to medical or non-medical reasons [2,3]. 

Passive therapy with polyclonal antibodies (pAb) has been used to treat various bacterial and viral diseases [4,5,6,7,8] and is also a potential tool to combat the COVID-19 pandemic [9]. An excellent animal model to evaluate pAb and other COVID-19 therapeutics are transgenic Tg(K18hACE2)2Prlmn/J (K18hACE2 transgenic) mice. Originally developed to study -CoV infection [10], these animals express human ACE2 (angiotensin converting enzyme 2) receptor primarily in the epithelial cells of internal organs such as brain, airways, liver, kidney, and gastrointestinal tract, and show many features of human SARS-CoV-2 infection including lethality [11,12]. Here we describe the pharmacokinetic (PK) and pharmacodynamic effects of COVID-HIGIV, a pAb hyperimmune product derived from human plasma enriched for anti-SARS-CoV-2 antibodies, in healthy wild-type C57Bl/6 mice and SARS-CoV-2 infected K18hACE2 transgenic mice.

## 2. Materials and Methods

COVID-HIGIV is a purified human IgG product manufactured by Emergent BioSolutions Canada Inc (Winnipeg, Canada). The immunoglobulin fraction of plasma from convalescent donors previously infected with SARS-CoV-2 collected by blood/plasma collection establishments was obtained using a scalable (200 to 1000 L plasma), established manufacturing process which includes anion-exchange chromatography and two orthogonal virus removal steps (filtration and solvent/detergent treatment). COVID-HIGIV used was a 10g% solution with a total protein concentration of 100 mg/mL (lot # PD_740_POC_17_001_006) and a neutralizing potency of 763 AU/mL.

The institutional animal care and use committee at Lovelace Biomedical Research Institute approved animal study protocols for the work presented here, as did the U.S. Army Medical Research and Development Command’s Animal Care and Use Review Office. Studies were not blinded. The number of animals used for each time point was typical for PK studies with a serial sacrifice design. Animals were quarantined for seven days before the study, and were randomized to sacrifice time points based on body weight. All procedures in infected animals were carried out within ABSL-3 (animal biosafety level 3) at Lovelace Biomedical Research Institute. 

Forty-two (gender-balanced; 8–10-week-old) wild-type C57Bl/6 mice were administered a single dose of 400 mg/kg (3052 AU/kg) COVID-HIGIV intravenously (i.v.) to the tail vein. Six animals (gender-balanced) were sacrificed at each of seven time points (1, 6, 24, 48, 120, 240, and 360 h) after COVID-HIGIV administration for PK analysis. Terminal blood was collected by cardiopuncture and processed to serum for analysis.

Forty-eight (gender-balanced; 8–12-week-old) B6.Cg-Tg(K18-ACE2)2Prlmn/J transgenic mice (C57Bl/6 background; RRID: IMSR_JAX:034860) were infected intranasally (i.n.) with 4.7 × 10^3^ TCID_50_ SARS-CoV-2 (strain US_WA1/2020; BEI Resources/NR-52281). Immediately (within a few minutes) after infection, forty-two animals were administered i.v. with a single dose of 400 mg/kg (3052 AU/kg) COVID-HIGIV and terminal samples were collected for PK analysis as described for C57Bl/6 mice above. The remaining six animals were untreated and served as virology controls. Animals were monitored for survival up to the time of scheduled sacrifice or, in the case of virology controls, up to 10 days post-infection (dpi). Body weights were collected one day before infection and at 3, 6, and 10 dpi. For virology control animals that succumbed before 10 dpi, bodyweights were collected on the day of humane euthanasia or upon being found dead. Lungs were also collected at the time of scheduled sacrifice, or in the case of untreated controls that succumbed before 10 dpi, at the time of humane euthanasia or upon being found dead. Terminal blood samples were collected and processed to serum for serum antibody analysis at the time of scheduled sacrifice. Gross necropsies were performed on all animals, and any abnormal findings were recorded.

For virus titration, lung tissues were homogenized in phosphate buffered saline and clarified of debris by centrifugation. Supernatants were serially diluted 10-fold in infection medium, and 0.1 mL of each dilution was used to inoculate VeroE6 cell monolayers at ≥90% confluency in five replicates. Plates were incubated for 72 h at 37 ℃ and 5% CO_2_ and examined for presence of cytopathic effect. The TCID_50_ titers were calculated using the Reed–Muench method [13].

Serum antibody assays were conducted at Emergent BioSolutions. All samples from both C57Bl/6 and K18hACE2 transgenic mice were sterilized by UV cross-linking at constant energy of 5000 J/cm^2^ for 10 min. In the case of samples from K18hACE2 transgenic mice this occurred before samples were transferred out of the ABSL-3 facility. Sterilized serum samples were tested using a MagPix binding assay derived from a validated test method and using a reference standard whose titer was established using a wild-type SARS-CoV-2 neutralization assay to estimate free anti-SARS-CoV-2 antibody titers reported in neutralizing units (AU) per mL. While measurement of anti-viral titers with a wild-type or pseudovirus neutralization assay is well accepted, binding assay titers have been shown to correlate with live virus neutralization assay [14,15,16,17].

Insufficient serum was collected from two male animals (one from each of the 1-h and 6-h timepoints) for testing; therefore, these timepoints had five samples assayed only. 

Data analyses were conducted in ‘R (4.0.2)’. No data points were excluded from analysis. Non-compartmental pharmacokinetic analyses were performed using the serial sampling design algorithm from the ‘PK (1.3-5)’ package, comparison of survival between control and COVID-HIGIV treated animals was conducted using the Kaplan-Meier log-rank test from the ‘survival (3.2-7)’ and ‘survminer (0.4.9)’ packages. Body weights were normalized as a percentage of body weights measured on the day prior to infection.

## 3. Results

**Figure 1:**
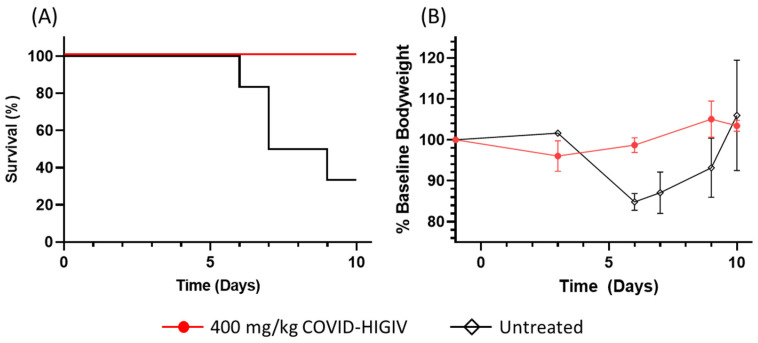
COVID-HIGIV enhances survival and reduces morbidity in SARS-CoV-2 infected transgenic K18hACE2. mice. K18hACE2 transgenic mice were infected with 4.7 × 10^3^ TCID_50_ SARS-CoV-2 i.n and monitored for survival for ten days. (**A**) A significant (*p* = 0.018) increase in survival was observed in animals given 400 mg/kg (3052 AU/kg) COVID-HIGIV i.v. (red) immediately after infection compared to virology control animals which were untreated (black). (**B**) Transient weight loss characteristic of SAR-CoV-2 infection in this model was reduced in animals given COVID-HIGIV. Values shown are averages ± standard error of the mean.

SARS-CoV-2 infection was lethal in untreated controls, with significant mortality (67%; 4 of 6 animals) observed at 10 dpi. In contrast, 100% of animals given COVID-HIGIV immediately after infection survived to scheduled sacrifice, including the six animals sacrificed at 10 dpi. This enhanced survival at 10 dpi with a single i.v. dose of COVID-HIGIV was statistically significant (*p* = 0.018) (Figure 1A). Morbidity in the form of body weight loss was also reduced with a peak weight loss of 4% at 3 dpi in COVID-HIGIV treated animals compared to a peak weight loss of 15% at 6 dpi in untreated animals (Figure 1B). Weight loss in the two untreated animals which survived was transient, with recovery to baseline occurring from 7 dpi to 10 dpi. 

Lungs of untreated virology control animals showed dark red discoloration, which was minimal in one animal, mild in four animals and marked in one animal. Similar, mild discoloration was observed in only three COVID-HIGIV treated animals, two that were sacrificed at 5 dpi and one that was sacrificed at 10 dpi. Median viable virus titers in lung of COVID-HIGIV treated animals were 1.53 × 10^6^ TCID_50_/g at 1 dpi, reducing to 1.44 × 10^3^ TCID_50_/g at 5 dpi and were undetectable at 10 dpi. Median viable virus titers in untreated virology controls at 6 and 7 dpi were 5.7 × 10^5^ TCID_50_/g and 5.1 × 10^5^ TCID_50_/g, respectively. Virus titers were undetectable in the untreated animal that succumbed on 9 dpi and the two untreated animals which underwent scheduled sacrifice at 10 dpi. Due to the limited time points available for the virology controls and the absence of matching COVID-HIG treated animals at moribund time points, statistical comparison between groups could not be conducted. 

**Figure 2:**
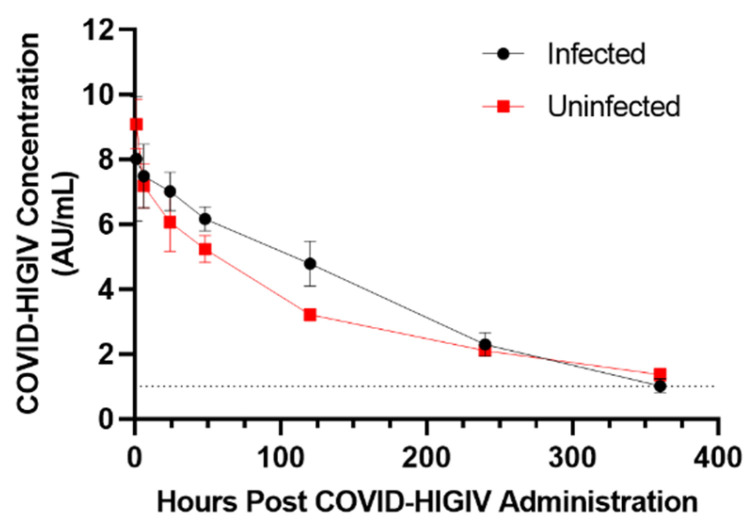
Pharmacokinetic profile of COVID-HIGIV in healthy and SARS-CoV-2 infected mice. Healthy C57bl/6 mice (red) were administered 400 mg/kg (3052 AU/kg) COVID-HIGIV i.v. and samples collected by serial sacrifice from 1 to 360 h after administration. Transgenic K18hACE2 transgenic mice (black) were identically treated but were infected with 4.7 × 10^3^ TCID_50_ SARS-CoV-2 (strain US_WA1/2020) i.n. immediately after COVID-HIGIV administration. Each data point represents the mean ± SEM of five or six individual animals.

Serum antibody concentrations over time in infected animals were similar to those observed in uninfected animals (Figure 2). Tmax was identical, and Cmax, AUC_0–∞_, and Cl were comparable, in infected and uninfected animals. In contrast, AUC_0–last_ was increased by 24.4% in infected animals compared to uninfected animals, while mean residence time (MRT), T_1/2_, and Vd were reduced by 35% to 40% (Table 1).

## 4. Discussion

In the current study, we demonstrated the efficacy of COVID-HIGIV in a lethal model of SARS-CoV-2 infection and established the pharmacokinetics of COVID-HIGIV in healthy wild-type mice and SARS-CoV-2 infected transgenic mice. 

Treatment with COVID-HIGIV as a single dose of 400 mg/kg (3052 AU/kg) was highly effective in preventing mortality in K18hACE2 transgenic mice, providing complete protection against SARS-CoV-2 infection. In contrast, as has been reported by others [11,12], SARS-CoV-2 infection was lethal in untreated controls, with two thirds of animals scheduled to be sacrificed at 10 dpi succumbing before the end of the study. Similarly, COVID-HIGIV treatment largely prevented morbidity in the form of body weight loss, whereas untreated controls showed substantial weight loss at 6 dpi and 7 dpi. The weight loss was transient in untreated controls that survived. The frequency of discoloration of lung tissues at necropsy was also reduced in the COVID-HIGIV treated animals. This efficacy is consistent with previous observations of reduced morbidity and pathology in hamsters treated with COVID-HIGIV [18] and in hamsters and K18-hACE2 transgenic mice treated with other antibody products [19,20,21]. However, due to a small sample size for the pharmacological aspects of this work, it is difficult to make a definitive conclusion on the efficacy of COVID-HIGIV in this model.

The serum antibody-time profiles of COVID-HIGIV were generally similar in healthy and infected animals, with somewhat increased AUC_0–last_ and somewhat reduced MRT, T_1/2_, and Vd in infected animals compared to healthy animals. This was unexpected, as it was presumed that antibody consumption resulting from neutralization of SARS-CoV-2 would lead to reduced circulating COVID-HIGIV concentrations. The similarities of COVID-HIGIV serum concentrations in infected an uninfected animals may be attributable to the absence of SARS-CoV-2 in systemic circulation, which is characteristic of this model [12]. Viral neutralization may be localized to the site of infection in the lung, with circulating antibodies having limited bioavailability per unit time in these tissues due to physiological constraints [22,23,24]. This would result in only relatively small effects on antibody consumption through viral neutralization, with little if any impact on the circulating concentrations of COVID-HIGIV. In addition, it is not completely understood if the mortality in this model is due to the collapse of the respiratory system, or due to brain infection, or both [11,12]. However, COVID-HIGIV was highly effective in protecting the infected K18hACE2 transgenic mice from death. 

Overall, these data show that the COVID-HIGIV PK in healthy and infected animals is comparable, and COVID-HIGIV is an effective treatment for SARS-CoV-2 infection.

## Figures and Tables

**Table 1:** Calculated mean ± standard error of the mean for pharmacokinetic parameters of COVID-HIGIV in healthy C57bl/6 mice and SARS-CoV-2 infected K18hACE2 transgenic mice.

Parameter	Healthy Mice	Infected Mice
AUC_0–last_ (AU·h/mL)	1126.72 ± 38.98	1401.16 ± 70.81
AUC_0–∞_ (AU·h/mL)	1454.54 ± 196.27	1544.06 ± 90.47
C_max_ (AU/mL)	9.08 ± 0.76	8.02 ± 1.92
T_max_ (h)	1.00 ± 0.00	1.00 ± 0.00
MRT (h)	233.30 ± 70.18	155.48 ± 15.63
T_1/2_ (h)	161.71 ± 48.64	107.77 ± 10.83
Cl (mL/h·kg)	2.10 ± 0.28	1.98 ± 0.12
V_d_ (mL)	489.52 ± 85.08	307.32 ± 25.74

## Data Availability

All data generated or analyzed during this study are included in this published article. The COVID-HIGIV product may be requested from the corresponding author for independent evaluation.
